# A key time point for cell growth and magnetosome synthesis of *Magnetospirillum gryphiswaldense* based on real-time analysis of physiological factors

**DOI:** 10.3389/fmicb.2013.00210

**Published:** 2013-07-24

**Authors:** Jing Yang, Shuqi Li, Xiuliang Huang, Tao Tang, Weizhong Jiang, Tongwei Zhang, Ying Li

**Affiliations:** ^1^Key Laboratory of Agro-biotechnology and Key Laboratory of Soil Microbiology, Ministry of Agriculture, College of Biological Sciences, China Agricultural UniversityBeijing, China; ^2^France-China Biomineralization and Nano-structure LaboratoryBeijing, China; ^3^College of Water Resources and Civil Engineering, China Agricultural UniversityBeijing, China; ^4^Institute of Geology and Geophysics, Chinese Academy of SciencesBeijing, China

**Keywords:** *Magnetospirillum gryphiswaldense*, submerged culture, physiological features, magnetosome synthesis, key time point

## Abstract

Pure culture of magnetotactic bacteria with high magnetosome yield has been achieved for only a few strains. The major obstacles involve the nutritional requirements and culture conditions of the cells. To increase cell density and magnetosome production, it is necessary to elucidate the physiological characteristics of a particular strain during cell growth and develop an appropriate artificial control strategy. Large-scale culture of *Magnetospirillum gryphiswaldense* strain MSR-1 was successfully performed for 48 h in a 42-L autofermentor, and several key physiological parameters were measured in real time. Maximal values of cell density (OD_565_) (19.4) and cell yield (dry weight) (4.76 g/L) were attained at 40 h. The key time point for cell growth and magnetosome formation was found to be 18–20 h. At this point, cells entered the log phase of growth, the maximal values of C_mag_ (1.78), iron content (0.47%), and magnetosome number (26 ± 3 per cell) were observed, superoxide dismutase (SOD) activity began to decrease more rapidly, ATP content dropped to an extremely low level (0.17 fmol), and reducing power (NADH/NAD^+^ ratio) began to increase very rapidly. Excessive levels of dissolved oxygen (≥20 ppb) and lactic acid in the medium caused notable cytotoxic effects after 20 h. Artificial control measures for fermentation must be based on realistic cell physiological conditions. At the key time point (18–20 h), cell density is high and magnetosomes have matured. The process of magnetosome synthesis involves a high consumption of ATP and reducing power, and the cells require replenishment of nutrients prior to the 18–20 h time point. Culture conditions that effectively minimize dissolved oxygen accumulation, lactic acid content, and reducing power at this point will enhance magnetosome yield without obvious inhibition of cell growth.

## Introduction

Magnetotactic bacteria (MTB) are a group of aquatic microbes characterized by the ability to orient along magnetic field lines based on the presence of intracellular nano-sized “magnet needles” termed magnetosomes. Magnetosomes are membrane-bound magnetite (Fe_3_O_4_) or greigite (Fe_3_S_4_) crystals that form one or multiple chains within the cell (Jogler and Schüler, 2009; Komeili, 2012). Because of their narrow size distribution (30–120 nm) and uniform morphology, magnetosomes have potential applications in magnetic separation techniques, diagnostics, and analytic detection (Matsunaga et al., 2004; Pollithy et al., 2011). All MTB known to date are difficult to culture because of their strict requirements in terms of nutrition, oxygen, and redox potential (Zhang et al., 2010; Zhu et al., 2010). Most known MTB are obligatory anaerobic, facultative anaerobic, or microaerobic. Therefore, only a small proportion of MTBs can be axenically isolated and cultured, and the obtainable cell densities do not allow the purification of sufficient numbers of magnetosomes for industrial applications (Jogler and Schüler, 2009). Improved methods are needed to increase MTB cell density and magnetosome yield at a reasonable cost.

*Magnetospirillum gryphiswaldense* MSR-1 is an MTB strain that has been the subject of considerable genetic research and can be cultured at high densities relative to other MTB (Jogler and Schüler, 2009). In a 2008 study, we achieved MSR-1 fermentation cell density (OD_565_) 7.24, cell yield 2.17 g/L, and magnetosome yield 41.7 mg/L (Sun et al., 2008). In 2010, we achieved cell density 12.0 and magnetosome yield 83.23 mg/L (Liu et al., 2010). In 2011, using an improved strategy for high-density culture of MSR-1 and large-scale magnetosome production through semicontinuous culture and reduction of osmotic factors that tend to inhibit cell growth, we achieved cell yield 9.16 g/L and magnetosome yield 356.52 mg/L (Zhang et al., 2011). In each of these studies, we focused on optimization of culture medium, adjustment of oxygen level and pH, and addition of nutrients, but did not carefully monitor cell physiological indicators. Increased knowledge of the physiological characteristics of the dynamic cell growth process will be helpful. *i.e*., better understanding of the physiology and growth principles of MTBs will allow us to reduce the effects of specific physiological inhibitors and further increase magnetosome yield. By analyzing cells cultured under physiological conditions, we can elucidate realistic principles of MSR-1 growth and develop improved stability control strategies.

We report here new comprehensive findings on MSR-1 culture and cell physiological features during submerged culture in a 42-L autofermentor. In preliminary experiments, we evaluated a variety of physiological parameters, including growth curves, magnetism [C_mag_ value, ratio of maximal and minimal scattering intensities (Schüler et al., 1995)], dissolved oxygen (dO_2_) and lactic acid (LA) levels in medium, iron content, ATP content, reducing power, and superoxide dismutase (SOD) activity in cells. Striking changes in most of these parameters were observed between hours 18–20 of culture, indicating a key time point for MSR-1 cell growth and magnetosome synthesis. The decreased levels of dO_2_, LA, and reducing power occurring at this key time point can be exploited for the improvement magnetosome yield without obvious inhibition of cell growth. This study is the first to utilize physiological parameters for the evaluation of MSR-1phenotype during fermentation. Our findings may be applicable to the culture of other MTB and microaerobic bacteria.

## Materials and methods

### Submerged culture of cells in a 42-L autofermentor

*M. gryphiswaldense* strain MSR-1 (DSM6361) was purchased from Deutsche Sammlung von Mikroorganismen und Zellkulturen (Brunswick, Germany). Seed culture was performed as described previously (Liu et al., 2010). The 42-L fermentor was filled with 30 L of medium containing 1.5 g sodium thioglycolate, 6 g magnesium sulfate heptahydrate, 15 g yeast extract, 6 g peptone, and 15 mL Wolfe's mineral solution. Feed medium contained 4.2 g ferric citrate, 129 g sodium lactate, 52.6 g LA, and 54.9 g ammonium chloride in a volume of 700 mL. MSR-1 cells were continuously cultured in the fermentor for 48 h, at which point the OD_565_ value began to decrease following a long-term rise. The temperature and pH were controlled to 30°C and 6.8, respectively, during culture. The pH was adjusted via the feed medium. The dO_2_ concentration was recorded using two probes that had different purposes. One probe was used for relative measurement (percent % as unit of data) and had lower accuracy. The other probe was used for absolute measurement (ppb as unit of data) and had higher accuracy. Calibration prior to measurements was required for the former probe but not for the latter. Before inoculation, we maintained an initial stirring rate (120 rpm) and airflow rate (0.94 L/min) for 2 h to ensure that oxygen was at saturation level in the medium. We then calibrated the dO_2_ as closely as possible to 100% (there were occasional minor fluctuations). The various study parameters were recorded after inoculation as functions of time (Table 1).

**Table 1:** **Adjustment of dissolved oxygen (dO_2_) and corresponding values of OD_565_ and C_mag_ during submerged culture of MSR-1 cells**.

**Time (h)**	**Stirring rate (rpm)**	**Airflow rate**	**dO_2_**		**OD_565_**	**C_mag_**
		**(L/min)**	**(%)**	**(ppb)**		
0	120	0.94	102.70	2451	0.1	0.74
8	120	0.95	0.00	12	0.3	0.24
10	140	1.45	0.00	4	0.4	0.80
12	160	1.95	0.00	4	0.7	1.39
14	180	1.97	0.00	3	1.1	1.38
16	180	1.95	0.00	2	1.5	1.65
18	200	1.95	0.00	3	1.7	1.52
20	230	2.95	0.00	6	2.1	1.78
22	250	3.98	0.00	4	3.2	1.65
24	270	3.96	0.00	17	4.8	1.71
26	280	3.99	0.00	35	6.3	1.38
28	290	3.98	0.00	9	8.3	1.50
30	300	3.97	0.00	215	10.8	1.54
32	300	1.97	0.00	186	13.7	1.68
34	300	1.95	0.00	204	14.6	1.41
36	300	1.95	12.40	661	16.8	1.43
38	300	1.95	21.60	962	18.1	1.20
40	300	1.95	29.80	1247	19.4	1.12

dO_2_ was measured using two probes with differing accuracies (see Materials and Methods). To maintain dO_2_ at the level suitable for magnetosome synthesis (0.0%), the stirring rate was changed at 10, 12, 14, 18, 20, 22, 24, 26, 28, and 30 h, and the airflow rate was changed at 10, 12, 20, 22, and 32 h.

Samples were taken every few hours for further analysis. After sampling, cell density (OD_565_) and magnetism (C_mag_) were measured immediately using the same spectrophotometer at wavelength 565 nm as described by Schüler et al. (1995). A 2-mL culture sample was centrifuged, and the LA concentration of the supernatant was measured immediately using a SBA-40C Biosensor analyzer (Institute of Biology, Shandong Academy of Sciences, China). Cells (in a defined volume) were washed twice with 50 mM Tris buffer (pH 7.0) and centrifuged at 4°C, and the cell pellets were stored at −80°C. Culture samples were suspended in an appropriate volume of sterile distilled water for subsequent measurements.

### Iron content

Another 2-mL culture sample was centrifuged in a 1.5-mL tube, and the pellet was dried at 60°C until it reached a constant weight. Iron content was measured by Inductively Coupled Plasma Optical Emission Spectrometry (ICP-OES; model Optima 5300DV, Perkin Elmer, Waltham MA, USA). The percentage of iron in cells was calculated as the iron content divided by the dry weight.

### ATP content

Another 2-mL culture sample was added with 10% trichloroacetic acid solution (1:1, v/v), incubated on ice for 10 min, and centrifuged for 5 min at 4°C. The ATP content of the supernatant was measured using an AF-100 ATP analyzer (DKK-TOA Corp., Tokyo, Japan). The ATP content of the sample was calculated as total ATP content / volume of sample/OD_565_ of sample.

### Reducing power

Reducing power was measured by a modification of the method of Perez et al. (2008). For NADH measurement, a 1-mL sample was incubated with 50 μL KOH (0.4 mol/L, pH 12.3) for 10 min at 30°C, centrifuged (12,000 rpm) for 10 min at 4°C, and the supernatant was assayed. For NAD^+^ measurement, a 1-mL sample was incubated with 125 μL HCl (0.4 mol/L, pH 1.3) for 10 min at 5°C, centrifuged (12,000 rpm) for 10 min at 4°C, and the supernatant was assayed. The reducing power was calculated as the ratio of NADH to NAD^+^.

### Transmission electron microscopy (TEM)

A culture sample was placed on a copper grid, washed twice with distilled water, dried, and observed with a transmission electron microscope (model JEM1230; JEOL, Tokyo, Japan).

### Superoxide dismutase activity

Cells were lysed by ultrasonication (ice bath, 50 W, 200 times, 50% duty cycle). Cell debris was removed by centrifugation at 4°C to yield a supernatant (used as crude enzyme extract), and the protein content (mg) was measured by Coomassie Brilliant Blue assay (Chial et al., 1993). The total SOD activity was measured using a SOD assay kit (Jiancheng Corp., Nanjing, China). The SOD activity of a sample was calculated as the total SOD activity divided by protein content.

## Results

### Precise control of dissolved oxygen concentration ensures optimal conditions for magnetosome formation

**Figure 1:**
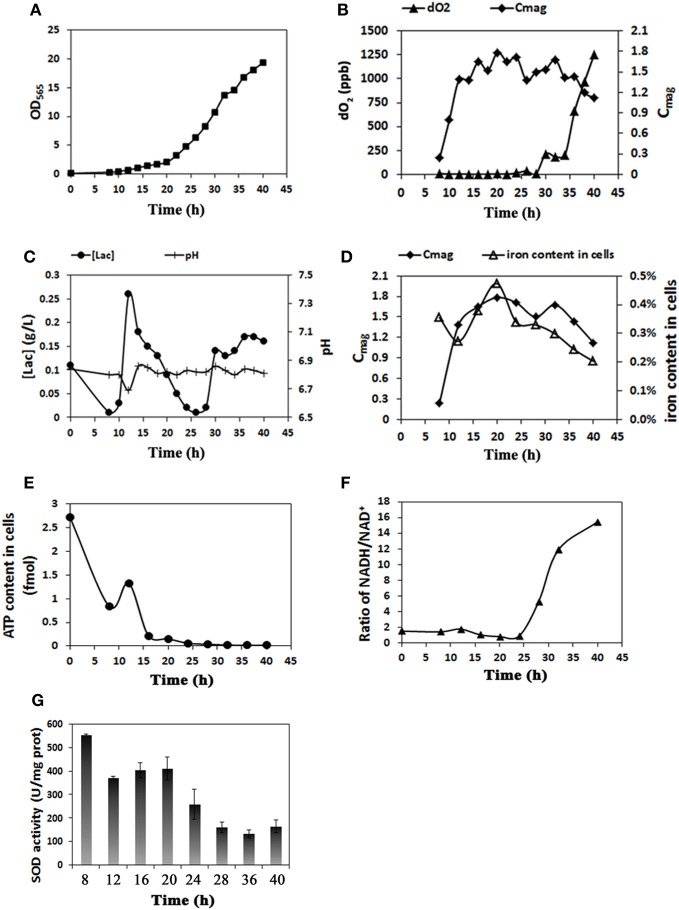
**Real-time physiological parameters of MSR-1 cells in submerged cultured in a 42 L autofermentor. (A)** Cell growth curve (OD_565_). Cells entered the log growth phase at 20 h and reached maximal OD at 40 h. **(B)** Comparison of dissolved oxygen (dO_2_) concentration and C_mag_ revealed that the optimal absolute dO_2_ concentration for magnetosome production is <20 ppb. **(C)** Lactic acid concentration ([lac]) and pH value were under coordinated control. **(D)** The level of iron content was closely related to that of C_mag_. **(E)** Cell ATP content decreased rapidly from 0 to 20 h and much more gradually thereafter. **(F)** The NADH/NAD^+^ ratio was low from 0 to 25 h and increased rapidly thereafter. **(G)** SOD activity assays at sampling times from 8 to 40 h. SOD activity was notably reduced after 20 h.

We performed several preliminary experiments to obtain stable results based on our past experience. Although there were slight differences in the data from different batches, the overall trends for cell growth rate and magnetosome yield were consistent (see Figures 1A, S1). The results from a representative experiment are shown in Figure 1 as an example. As showed in Figure 1, cells grew slowly (OD_565_ ≤ 2.0) until hour 20. Thereafter, cell growth entered the log phase. Maximal values of OD_565_ (19.4) (Figure 1A) and cell yield (dry weight) (4.76 g/L) were observed at hour 40. In MSR-1 culture, high dO_2_ levels (>1%) promote cell growth but inhibit magnetosome formation (Heyen and Schüler, 2003; Liu et al., 2010). To balance these conflicting effects, we set the initial (hour 0) stirring rate to 120 rpm, the airflow rate to 0.94 L/min and the saturated dO_2_ under these conditions to 100% (showed 102.7% after setting), based on the results of our previous study (Liu et al., 2010). After inoculation, the dO_2_ level decreased rapidly to 0.0% at 8 h (Table 1). During this period, cell density (OD_565_) increased 3-fold (from 0.1 at 0 h to 0.3 at 8 h), indicating a high oxygen requirement during the early stage of cell growth. To maintain a suitable dO_2_ (0.0%) for magnetosome formation, we subsequently altered the stirring rate at 2-h intervals between 10 and 30 h and altered the airflow rate at 10, 12, 20, 22, and 32 h (Table 1). To precisely control dO_2_ concentration, we used two probes with differing accuracies as described in Materials and Methods, one for relative dO_2_ measurement (percent %), the other for absolute measurement (ppb). This approach allowed us to accurately detect even tiny changes of dO_2_, particularly at extremely low concentrations. From 28 to 34 h, the absolute dO_2_ level gradually increased nearly 23-fold, from 9 to 204 ppb, while the probe used for relative measurement continuously showed a value below the limit of detection (0.0%), which may have caused a delay in adjusting the stirring rate and airflow rate in the fermentor (Table 1). A high dO_2_ concentration affected magnetosome formation. From 8 to 24 h, the absolute dO_2_ concentration remained <20 ppb, while the C_mag_ value increased from 0.24 at 8 h to 1.78 at 20 h and remained high until 24 h (Figure 1B). dO_2_ increased gradually from 17 ppb at 24 h to 1247 ppb at 40 h, while C_mag_ decreased from 1.71 at 24 h to 1.12 at 40 h. Taken together, these findings indicate that the optimal concentration of absolute dO_2_ for magnetosome production is <20 ppb.

### Lactic acid concentration reflects growth state

The LA feed medium contained LA, sodium lactate, ferric citrate, and ammonium chloride (see Materials and Methods). The LA in this medium was utilized both as a carbon source and a pH regulator. The supply of feed medium was auto-controlled via a computer to maintain a pH value of 6.8 throughout cell culture. The LA concentration in medium at each time point was recorded using a Biosensor analyzer and used as an indicator of the cell growth state. The LA concentration decreased rapidly from 0.26 g/L at 12 h to 0.01 g/L at 26 h, corresponding to the log phase of growth curve and reflecting the high cellular demand for LA as a source of carbon and energy for growth (Figure 1C). The LA concentration then increased from 0.01 g/L at 26 h to 0.16 g/L at 40 h. Excessive LA was present during this period and was associated with frequent pH fluctuations and rapid cellular metabolism (Figure 1C).

### The period 18–20 h is a key time point for iron metabolism and magnetosome production

**Figure 2:**
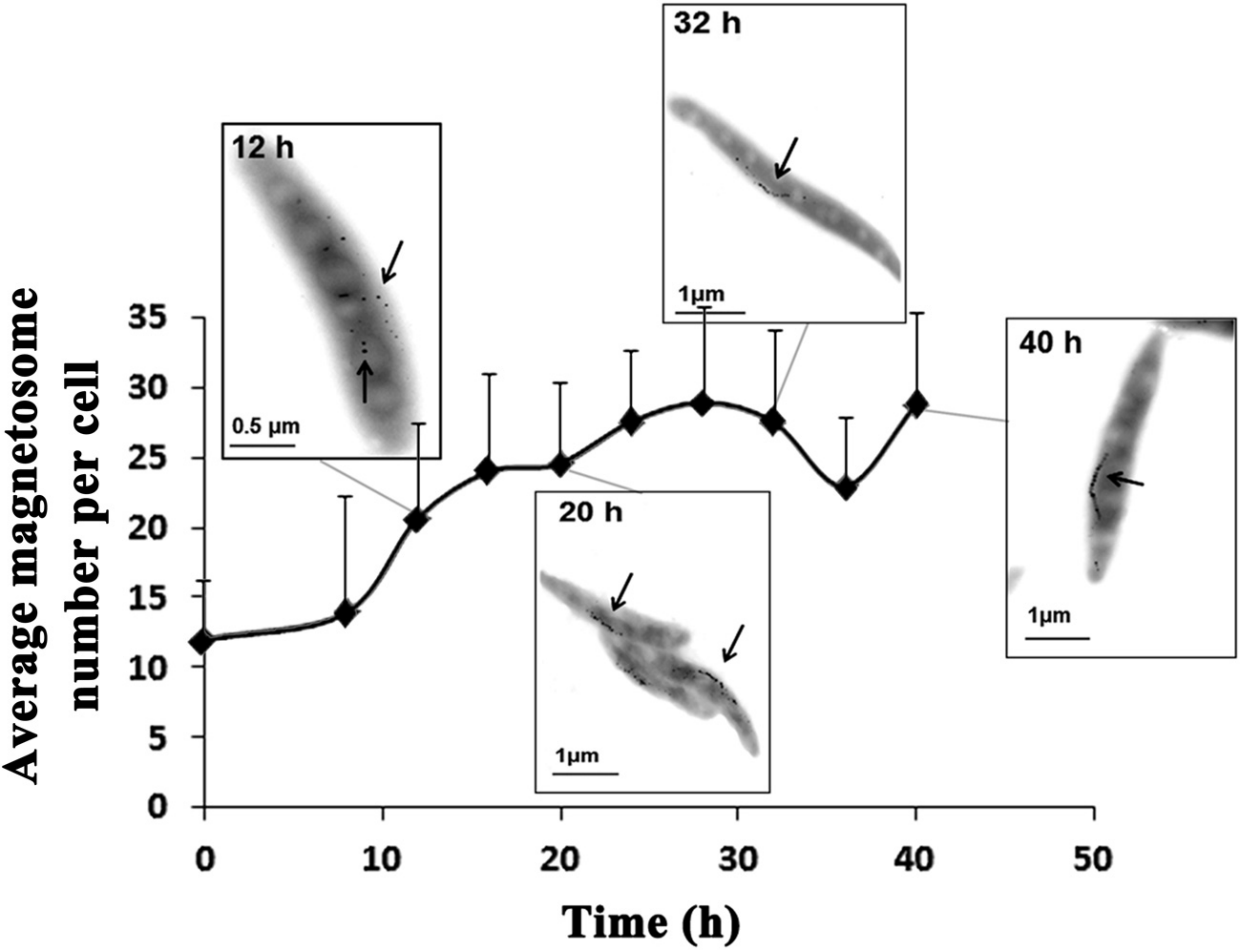
**Magnetosome number increased as a function of time.** Typical TEM photos taken at 12, 20, 32, and 40 h are shown. At 8 h, magnetosomes were scattered. From 20 to 40 h, magnetosome chains became completed and mature. Magnetosomes are indicated by arrows.

C_mag_ can be a useful parameter for estimating the magnetosome content of cells (Schüler et al., 1995), but it is based on an indirect method. A more direct method of measurement is based on TEM and the iron content of cells. The iron content in MTB can be 100-fold (or more) higher than in *E. coli* (Blakemore et al., 1979) and is concentrated primarily in magnetosomes (Kasama et al., 2006). We measured iron content by ICP-OES as described in Materials and Methods. C_mag_ and iron content both increased gradually from 8 to 20 h, reached maximal values (1.78 and 0.47%) at 20 h, and then decreased (to 1.12 and 0.20%, respectively) at 40 h (Figure 1D). TEM showed that the average number of magnetosomes per cell increased from 12 to 24 during the initial phase (0–20 h) and that magnetosome chains were not integrated at 12 h (Figures 2, S2 and Table 2). From 20 to 40 h, the average magnetosome number remained essentially constant (26 ± 3), and the chains were gradually integrated and matured (Figures 2, S2). We concluded that 20 h is an important transitional time point for magnetosome production. In view of the rapid decrease of LA content in medium around this time (Figure 1C), it is clearly important to provide sufficient nutritional supplements prior to 20 h to ensure the rapid growth of new cells.

**Table 2:** **Statistical processing of magnetosome number per cell at different time points**.

	**Sampling time points**										
	**Groups**	**0 h**	**8 h**	**12 h**	**16 h**	**20 h**	**24 h**	**28 h**	**32 h**	**36 h**	**40 h**
Percentage of magnetosome number per cell (%)	0–5	5.6	4.8	0.0	0.0	0.0	0.0	0.0	0.0	0.0	0.0
	6–10	44.4	42.9	10.0	0.0	3.4	0.0	0.0	0.0	0.0	0.0
	11–15	27.8	23.8	10.0	8.7	3.4	0.0	0.0	0.0	4.8	0.0
	16–20	16.7	23.8	25.0	26.1	24.1	4.3	10.0	13.0	38.1	12.5
	21–25	5.6	4.8	25.0	26.1	27.6	34.8	20.0	26.1	23.8	20.8
	26–30	0.0	0.0	25.0	21.7	31.0	30.4	40.0	13.0	19.0	16.7
	31–35	0.0	0.0	5.0	13.0	13.8	21.7	20.0	43.5	14.3	37.5
	36–40	0.0	0.0	0.0	4.3	0.0	8.7	10.0	4.3	0.0	12.5
AVE^*^		11.7	14.3	20.9	23.9	24.5	28.1	28.6	28.1	22.7	28.0
SD^**^		4.3	8.7	7.0	6.4	5.5	5.3	6.9	6.3	5.3	6.7

* Average magnetosome number per cell.

** Standard deviation.

### Magnetosome synthesis consumes a large amount of ATP and accumulates reducing power

The formation of magnetosomes in MTB is presumed to consume a large amount of energy. The availability of ATP as an energy source has been shown to be required for iron uptake in MTB (Nakamura et al., 1995; Schüler, 1999). We used an ATP analyzer to measure the ATP content of cells. From 0 to 20 h, the period of magnetosome synthesis, energy (i.e., ATP content) in cells decreased rapidly from 2.73 to 0.17 fmol. From 20 to 40 h, the ATP content continued to decrease from 0.17 fmol to near zero (0.02 fmol); however, the rate of decrease was lower than that from 0 h to 20 h (Figure 1E).

NADH plays an important role in ATP generation in cells and provides a proton gradient across the inner mitochondrial membrane for ATP production by ATP synthetase (Bonora et al., 2012). We used a modified enzyme reduction method to measure reducing power (as the ratio NADH/NAD^+^) (Perez et al., 2008). In contrast to the trend for ATP, the NADH/NAD^+^ ratio remained low (≤ 2) and fluctuated slightly during the first 20 h (Figure 1F). Thereafter, as the cells entered the log phase of growth and the magnetosomes matured, the NADH/NAD^+^ ratio increased rapidly to 15 at 40 h. These findings indicate that the process of magnetosome synthesis consumes a large amount of ATP and accumulates reducing power.

### Superoxide dismutase activity decreases rapidly after 20 h

SOD in microbes helps remove the superoxide anion radical (O_2_^−^) and hydrogen peroxide (H_2_O_2_), which have destructive effects on cell macromolecules (Cabiscol et al., 2000). In MTB, SOD also reduces oxidative stress during magnetosome formation. We used a SOD assay kit to monitor SOD activity in cells. The activity declined gradually from 555 U/mg at 8 h to 410 U/mg at 20 h and then rapidly to 164 U/mg at 40 h (Figure 1G).

## Discussion

The development of improved methods for high-yield magnetosome production has been a major goal of MTB research for over a decade. Progress in this area has been achieved primarily through optimization of pH, temperature, redox potential, dO_2_ level, and growth medium and feed medium composition (Heyen and Schüler, 2003; Sun et al., 2008; Liu et al., 2010; Zhang et al., 2011). Our knowledge of the physiological characteristics of MTB during the dynamic process of cell growth remains fragmentary (Ban et al., 2010). Even the reason why the growth of several MTBs is promoted by trace quantities of peptone remains unclear (Heyen and Schüler, 2003; Zhu et al., 2010). We recently observed that sodium chloride acts as a physiological inhibitor of MSR-1 growth (Zhang et al., 2011). Further studies of physiological factors in MTB are needed.

We achieved successful large-scale culture of MSR-1 in a 42-L fermentor during 48 h in the present study. Maximal cell density and cell yield were 19.4 (OD_565_) and 4.76 g/L (dry weight), respectively. Higher values were attained in one of our previous studies (Liu et al., 2010). We measured the dynamic values of 9 key physiological parameters: growth curve, magnetism (C_mag_), dO_2_ and LA content in medium, iron content, magnetosome number, ATP content, reducing power, and SOD activity in cells. The period from 18 to 20 h was identified as a key time point for cell growth and magnetosome formation. During this period, cell growth entered its log phase, maximal cellular values of C_mag_ (1.78), iron content (0.47%), and magnetosome number (26 ± 3) were reached, SOD activity began to decline more rapidly, ATP content dropped to near-zero, and reducing power (NADH/NAD^+^ ratio) increased rapidly. To precisely control dO_2_, we performed measurements using two probes with differing accuracies. The optimal dO_2_ concentrations for magnetosome production were 0.0% (relative) and <20 ppb (absolute).

LA in feed medium is used as both a carbon source and pH regulator. From 12 to 26 h, the LA concentration decreased rapidly, corresponding to the log phase of growth and reflecting the high cellular demand for LA as a source of carbon and energy for growth. We therefore recommend that pure sodium lactate be added intermittently to the feed medium during this period to meet the requirements of the cells. From 26 to 40 h, the LA concentration increased greatly, resulting in excessive LA levels associated with frequent pH fluctuations and rapid cellular metabolism. Excessive levels of organic acids such as LA have been shown to inhibit cell growth (Pieterse et al., 2005); this effect may explain the gradual reduction of growth rate we observed. As an alternative to the use of LA, we can adjust the pH using other compounds (e.g., carbon dioxide) or by adding feed medium manually at later stages.

ATP is the major energy source in cells and is required for physiological processes such as cell metabolism, signal transduction, and molecular transportation. In MTB, ATP is also involved in iron uptake (Nakamura et al., 1995; Schüler, 1999)and inducing the polymerization of MamK (Mitraki et al., 2012), an actin-like protein involved in the alignment of magnetosome chains (Scheffel et al., 2005). Our findings demonstrate that magnetosome synthesis in MTB consumes large amounts of ATP. NADH plays an important role in generating ATP and provides a proton gradient across the inner mitochondrial membrane for ATP production by ATP synthetase (Bonora et al., 2012). In contrast to the usual trend of ATP change, NADH was accumulated during the fermentation process. We found previously that reducing power is significantly increased during magnetosome synthesis and that excessive reducing power suppresses magnetosome synthesis and cell growth in later periods (unpubl. data). A high NADH/NAD^+^ ratio indicates that little NADH is being used to supply protons, resulting in a low cellular ATP content. In MTB, excessive reducing power is consumed through polyhydroxybutyrate (PHB) synthesis and hydrogen release (Ban et al., 2010). This process may account in part for the fact that MTB contain many PHB granules (Schultheiss et al., 2005; Komeili, 2006). When PHB synthase gene was knocked out in MSR-1, the amount of magnetosome increased nearly 30% compared with wild-type strain (Liu et al., 2008). So, it seems that an energy competition exists between the PHB and magnetosome synthesis process.

H_2_O_2_ can form a hydroxyl radical (HO·) when it receives an electron from ferrous iron. HO· is the only reactive oxygen species that can directly damage most biomolecules (Imlay, 2003). In the present study, magnetosomes reached their maximal number at 20 h. At this point, reductions were observed for the intracellular iron content (Figure 1D) and the amount of SOD used to remove reactive oxygen species (Figure 1G). This could be explained as, in the late log phase, when dO_2_ and nutrients were enough, magnetosome formation and maturation maybe cannot catch up the cell dividing rate, resulting in diluted magnetosomes in cells, which was indicated by the C_mag_ trend (Figure 1D). Therefore, the reduced SOD activity may be caused similarly from diluted reactive oxygen species. Recent studies have shown that magnetosomes and artificial magnetic nanoparticles are able to scavenge reactive oxygen species (Gao et al., 2007; Guo et al., 2012). This ability may account in part for the observed decrease in SOD activity.

## Conclusion

Artificial manipulation of fermentation processes for maximizing yield must be based on realistic cell physiological conditions. We found that 18–20 h is a key time point for *M. gryphiswaldense* MSR-1 cell growth and magnetosome synthesis. At this point, cell density is high, magnetosomes have matured, and cells require replenishment of nutrients. Culture conditions that effectively minimize dO_2_ accumulation, LA content, and reducing power at this point will promote magnetosome yield without obvious inhibition of cell growth. Artificial culture of MTB requires precise control of dO_2_ at low concentrations (≤20 ppb) that are suitable for magnetosome synthesis. Future analytical transcriptome studies involving the forced expression of MSR-1 genes that are involved in the metabolism of ATP, NADH, oxygen and iron will help us elucidate the optimal conditions for MTB culture.

### Conflict of interest statement

The authors declare that the research was conducted in the absence of any commercial or financial relationships that could be construed as a potential conflict of interest.

## Supplementary material

FIGURE S1 **A set of representative preliminary fermention data: growth and C_mag_ curves.** Cell growth enters the log phase at 20 h, with a maximum OD_565_ value at 36 h. C_mag_ increased gradually from 8 to 20 h, reached maximal values at 20 h, maintaining for a little while, and then showing a little decrease. The overall trends of this preliminary data are the same with our formally addressed data.

FIGURE S2 **Statistical processing of the magnetosome number per cell at four sampling time points.** Horizontal axis: The eight classes that magnetosome numbers per cell are grouped in. Vertical axis: Percentage of magnetosome number among the eight classes. Ave: average magnetosome number per cell. SD: standard deviation.
