# Supplementary material for: A key time point for cell growth and magnetosome synthesis of Magnetospirillum gryphiswaldense based on real-time analysis of physiological factors
Source: Front Microbiol. 2013 Jul 24;4:210. doi: 10.3389/fmicb.2013.00210 (PMC3721002; doi:10.3389/fmicb.2013.00210)
Supplement: FIGURE S1 — A set of representative preliminary fermention data: growth and Cmag curves. Cell growth enters the log phase at 20 h, with a maximum OD565 value at 36 h. Cmag increased gradually from 8 to 20 h, reached maximal values at 20 h, maintaining for a little while, and then showing a little decrease. The overall trends of this preliminary data are the same with our formally addressed data. [file DataSheet1.DOCX]

**Supplementary figure1**

**
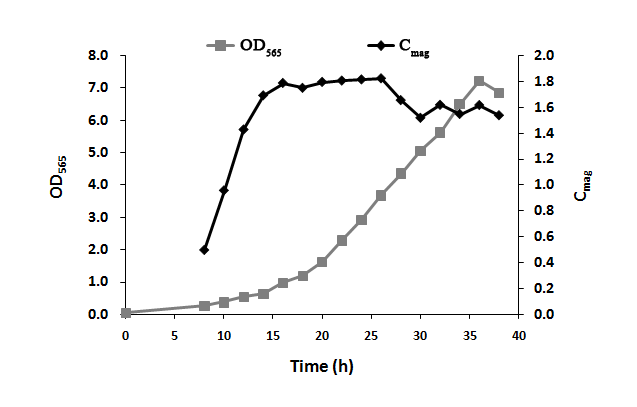
**

Figure S1 A set of representative preliminary fermention data: growth and C_mag_ curves. Cell growth enters the log phase at 20h, with a maximum OD_565_ value at 36h. Cmag increased gradually from 8 to 20 h, reached maximal values at 20 h, maintaining for a little while, and then showing a little decrease. The overall trends of this preliminary data are the same with our formally addressed data.
