# Supplementary material for: A key time point for cell growth and magnetosome synthesis of Magnetospirillum gryphiswaldense based on real-time analysis of physiological factors
Source: Front Microbiol. 2013 Jul 24;4:210. doi: 10.3389/fmicb.2013.00210 (PMC3721002; doi:10.3389/fmicb.2013.00210)
Supplement: FIGURE S2 — Statistical processing of the magnetosome number per cell at four sampling time points. Horizontal axis: The eight classes that magnetosome numbers per cell are grouped in. Vertical axis: Percentage of magnetosome number among the eight classes. Ave: average magnetosome number per cell. SD: standard deviation. [file DataSheet2.DOCX]

**Supplementary figure2**


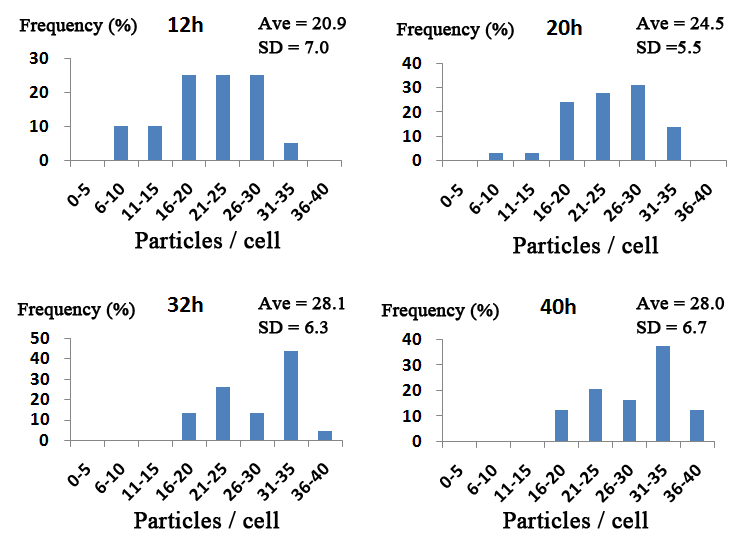


Figure S2 Statistical processing of the magnetosome number per cell at four sampling time points. Horizontal axis: The eight classes that magnetosome numbers per cell are grouped in. Vertical axis: percentage of magnetosome number among the eight classes. Ave: average magnetosome number per cell. SD: standard deviation.
